# Chlorido{4,4′,6,6′-tetra-*tert*-butyl-2,2′-[*o*-phenyl­enebis(nitrilo­methyl­idyne)]diphenolato-κ^4^ *O*,*N*,*N*′,*O*′}manganese(III)

**DOI:** 10.1107/S1600536809050314

**Published:** 2009-11-28

**Authors:** Naser Eltaher Eltayeb, Siang Guan Teoh, Chin Sing Yeap, Hoong-Kun Fun, Rohana Adnan

**Affiliations:** a School of Chemical Science, Universiti Sains Malaysia, 11800 USM, Penang, Malaysia; b X-ray Crystallography Unit, School of Physics, Universiti Sains Malaysia, 11800 USM, Penang, Malaysia

## Abstract

The asymmetric unit of the title Schiff base complex, [Mn(C_36_H_46_N_2_O_2_)Cl], comprises two crystallographically independent mol­ecules. The Mn^III^ centre in each mol­ecule adopts a distorted square-pyramidal geometry. Each Mn^III^ ion is coordinated by the N_2_O_2_ atoms of the tetra­dentate Schiff base ligand forming the basal plane and the coordinated chloride anion occupies the apical position. Four bifurcated intra­molecular C—H⋯O contacts stabilize the mol­ecular structure. In the crystal packing, mol­ecules are linked into dimers *via* inter­molecular C—H⋯Cl contacts and further stabilized by C—H⋯π inter­actions. The crystal studied was a non-merohedral twin, the refined ratio of the twin components being 0.441 (1):0.559 (1).

## Related literature

For biological applications of Schiff base derivatives, see: Dixit & Srinivasan (1988 ▶); Glatzel *et al.* (2004 ▶); Lu *et al.* (2006 ▶); Stallings *et al.* (1985 ▶). For a related structure, see: Eltayeb *et al.* (2007 ▶). For the stability of the temperature controller used for the data collection, see: Cosier & Glazer (1986 ▶).
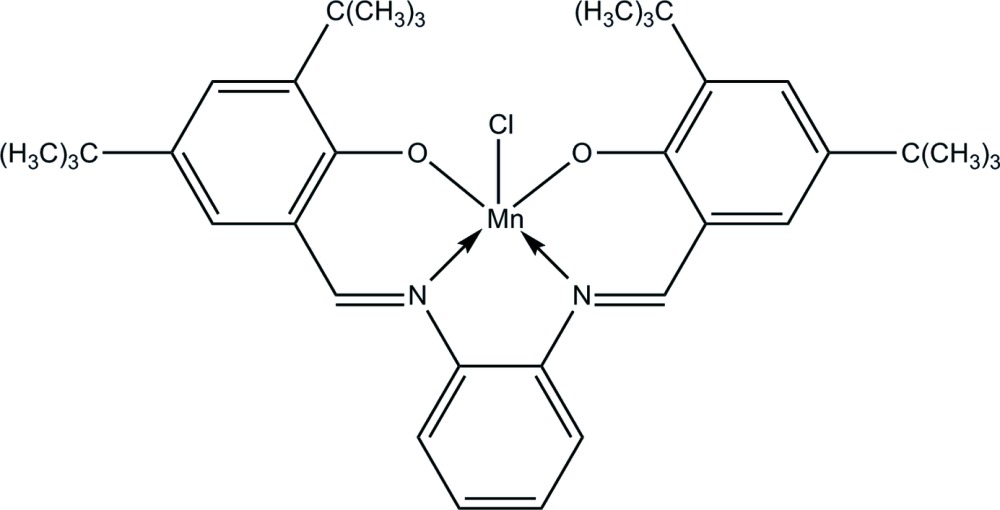


## Experimental

### 

#### Crystal data

[Mn(C_36_H_46_N_2_O_2_)Cl]
*M* *_r_* = 629.14
Triclinic, 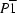

*a* = 10.7650 (6) Å
*b* = 16.8997 (9) Å
*c* = 20.2324 (11) Å
α = 107.357 (3)°
β = 90.010 (3)°
γ = 107.294 (3)°
*V* = 3338.4 (3) Å^3^
*Z* = 4
Mo *K*α radiation
μ = 0.51 mm^−1^
*T* = 100 K
0.40 × 0.32 × 0.14 mm

#### Data collection

Bruker SMART APEXII CCD area-detector diffractometer
Absorption correction: multi-scan (**SADABS**; Bruker, 2005 ▶) *T*_min_ = 0.822, *T*_max_ = 0.931
15265 measured reflections
15265 independent reflections
12633 reflections with *I* > 2σ(*I*)

#### Refinement

*R*[*F* ^2^ > 2σ(*F* ^2^)] = 0.049
*wR*(*F* ^2^) = 0.128
*S* = 1.07
15265 reflections
764 parameters
H-atom parameters constrained
Δρ_max_ = 0.90 e Å^−3^
Δρ_min_ = −0.87 e Å^−3^

### 

Data collection: *APEX2* (Bruker, 2005 ▶); cell refinement: *SAINT* (Bruker, 2005 ▶); data reduction: *SAINT*; program(s) used to solve structure: *SHELXTL* (Sheldrick, 2008 ▶); program(s) used to refine structure: *SHELXTL*; molecular graphics: *SHELXTL*; software used to prepare material for publication: *SHELXTL* and *PLATON* (Spek, 2009 ▶).

## Supplementary Material

Crystal structure: contains datablocks global, I. DOI: 10.1107/S1600536809050314/tk2582sup1.cif

Structure factors: contains datablocks I. DOI: 10.1107/S1600536809050314/tk2582Isup2.hkl

Additional supplementary materials:  crystallographic information; 3D view; checkCIF report

## Figures and Tables

**Table 1:** Hydrogen-bond geometry (Å, °)

*D*—H⋯*A*	*D*—H	H⋯*A*	*D*⋯*A*	*D*—H⋯*A*
C7*A*—H7*A*⋯Cl1*A* ^i^	0.93	2.81	3.648 (3)	151
C23*A*—H23*A*⋯O1*A*	0.96	2.32	2.963 (4)	124
C24*A*—H24*A*⋯O1*A*	0.96	2.38	3.021 (4)	124
C31*A*—H31*B*⋯O2*A*	0.96	2.25	2.914 (3)	125
C32*A*—H32*C*⋯O2*A*	0.96	2.46	3.079 (4)	122
C14*B*—H14*B*⋯Cl1*B* ^ii^	0.93	2.79	3.624 (3)	150
C23*B*—H23*E*⋯O1*B*	0.96	2.35	2.994 (4)	124
C24*B*—H24*F*⋯O1*B*	0.96	2.33	2.982 (4)	125
C31*B*—H31*D*⋯O2*B*	0.96	2.40	3.035 (4)	124
C32*B*—H32*F*⋯O2*B*	0.96	2.29	2.957 (4)	126
C28*A*—H28*B*⋯*Cg*1^iii^	0.96	2.70	3.658 (4)	174
C36*B*—H36*D*⋯*Cg*2^iv^	0.96	2.64	3.597 (4)	174

Symmetry codes: (i) 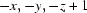; (ii) 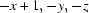; (iii) 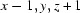; (iv) 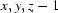. *Cg*1 and *Cg*2 are centroids of the benzene rings C1*A*–C6*A* and C15*B*–C20*B*, respectively.

**Fig. 1.**
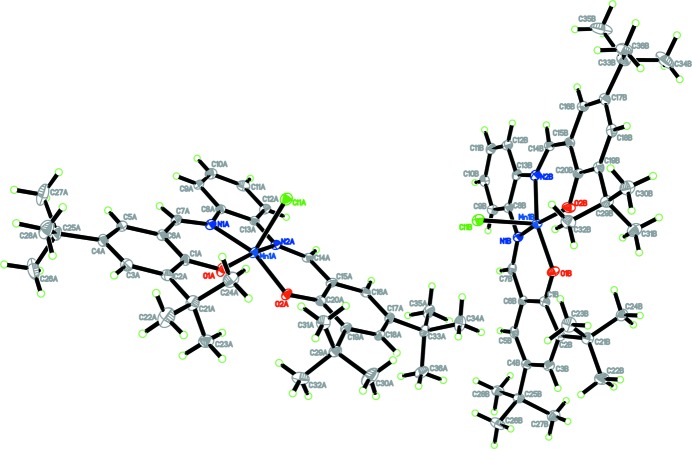
The molecular structure of the two independent molecules in title compound with 50% probability ellipsoids for non-H atoms.

**Fig. 2.**
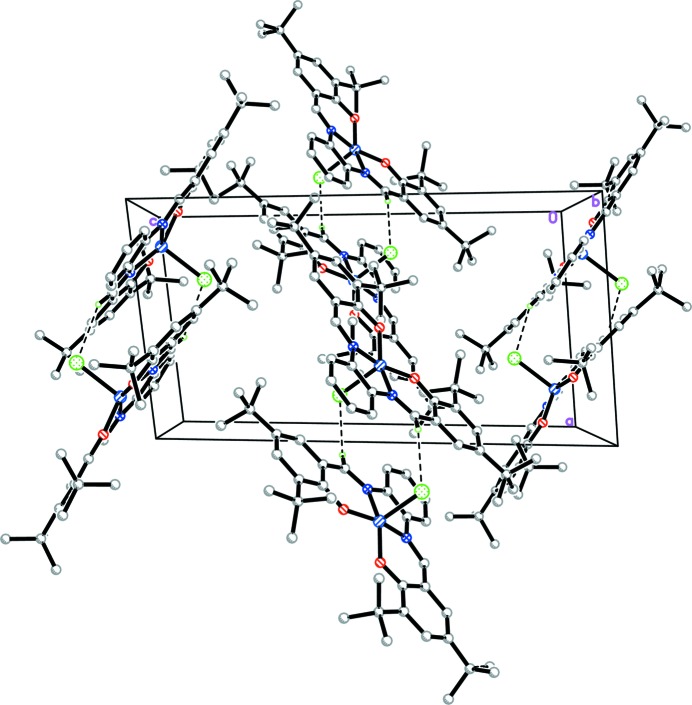
The crystal packing in the title compound, viewed down the b axis, showing the molecules are linked into dimers. Intermolecular hydrogen bonds are shown as dashed lines.

## supplementary crystallographic information

### Comment

Manganese complexes with Schiff base ligands have attracted considerable interest in the past decades and recently, due to their variety of applications in chemistry, biology, physics and advanced materials. It had been used as a model for the oxygen-evolving complex of photosystem II (Glatzel *et al.*, 2004), catalysis (Dixit & Srinivasan, 1988), single-molecule magnet (Lu *et al.*, 2006), and it serves as a model for the active sites of manganese-containing metal enzymes (Stallings *et al.*, 1985). Previously, we reported the crystal structure of (ethanol-*κO*){4,4',6,6'-tetra-*tert*-butyl-2,2'-[1,2-phenylenebis(nitrilomethylidyne)]diphenolato-*κ*^4^*O*,*O*',*N*,*N*'}zinc(II) ethanol solvate (Eltayeb *et al.*, 2007). Herein, we report the crystal structure of chlorido{4,4',6,6'-tetra-*tert*-butyl-2,2'-[*o*-phenylenebis(nitrilomethylidyne)]diphenolato-*κ*^4^*O*,*O*',*N*,*N*'}manganese(III).

The asymmetric unit of title compound consists of two crystallographically independent molecules, *A* and *B*. The Mn^III^ ion in each molecule adopts a distorted square-pyramidal geometry which is coordinated by the N_2_O_2_ atoms of the tetradentate Schiff base ligand. The apex position is occupied by a chloride ion. The Mn1 ions in molecules *A* and *B* are displaced by 0.3388 (4) and 0.3351 (4) Å out of the mean N1/N2/O1/O2 basal planes towards the axial Cl1 atoms, respectively. The dihedral angles between the central benzene ring (C8–C13) and the other two benzene rings (C1–C6 and C15–C20) are 8.84 (14) and 5.02 (13)° for molecule A, respectively, whereas these angles are 9.74 (14) and 9.35 (14)°, respectively, in molecule B. The geometric parameters are comparable to the previously reported structure (Eltayeb *et al.*, 2007).

Four bifurcated intramolecular C—H···O hydrogen bonds stabilized the molecular structure (Table 1). In the crystal packing (Fig. 2), the molecules are linked into dimers *via* intermolecular C7A—H7A···Cl1B and C14B—H14B···Cl1A contacts and are further stabilized by C—H···π interactions (Table 1).

### Experimental

A sample of 3,5-di-*tert*-butyl-2-hydroxybenzaldehyde (0.936 g, 4 mmol) was added to a solution of *o*-phenylenediamine (0.216 g, 2 mmol) in ethanol (30 ml). The mixture was refluxed with stirring for half an hour. Then (0.394 g, 2 mmol) manganese chloride tetrahydrate in ethanol (10 ml) was added, followed by triethylamine (0.5 ml, 3.6 mmol). The mixture was refluxed at room temperature for 3 h. A brown precipitate was obtained, washed with ethanol (5 ml), dried, and then washed by copious amount of diethyl ether. Brown crystals were formed after three days of slow evaporation of diethyl ether solution of the complex held at room temperature.

### Refinement

All H atoms were positioned geometrically and refined using a riding model, with C–H = 0.93 or 0.96 Å and *U*_iso_(H) = 1.2 or 1.5 *U*_eq_(C). The rotating group model was applied for the methyl groups. The crystal studied was a non-merohedral twin with a refined BASF of 0.441 (1).

### Crystal data

**Table:**

[Mn(C_36_H_46_N_2_O_2_)Cl]	*Z* = 4
*M**_r_* = 629.14	*F*(000) = 1336
Triclinic, *P*1	*D*_x_ = 1.252 Mg m^−^^3^
Hall symbol: -P 1	Mo *K*α radiation, λ = 0.71073 Å
*a* = 10.7650 (6) Å	Cell parameters from 9816 reflections
*b* = 16.8997 (9) Å	θ = 2.1–30.4°
*c* = 20.2324 (11) Å	µ = 0.51 mm^−^^1^
α = 107.357 (3)°	*T* = 100 K
β = 90.010 (3)°	Block, brown
γ = 107.294 (3)°	0.40 × 0.32 × 0.14 mm
*V* = 3338.4 (3) Å^3^	

### Data collection

**Table:**

Bruker SMART APEXII CCD area-detector diffractometer	15265 independent reflections
Radiation source: fine-focus sealed tube	12633 reflections with *I* > 2σ(*I*)
graphite	*R*_int_ = 0.000
φ and ω scans	θ_max_ = 27.5°, θ_min_ = 1.3°
Absorption correction: multi-scan (*SADABS*; Bruker, 2005)	*h* = −13→13
*T*_min_ = 0.822, *T*_max_ = 0.931	*k* = −21→20
15265 measured reflections	*l* = 0→26

### Refinement

**Table:**

Refinement on *F*^2^	Primary atom site location: structure-invariant direct methods
Least-squares matrix: full	Secondary atom site location: difference Fourier map
*R*[*F*^2^ > 2σ(*F*^2^)] = 0.049	Hydrogen site location: inferred from neighbouring sites
*wR*(*F*^2^) = 0.128	H-atom parameters constrained
*S* = 1.07	*w* = 1/[σ^2^(*F*_o_^2^) + (0.0506*P*)^2^ + 1.5837*P*] where *P* = (*F*_o_^2^ + 2*F*_c_^2^)/3
15265 reflections	(Δ/σ)_max_ = 0.001
764 parameters	Δρ_max_ = 0.90 e Å^−^^3^
0 restraints	Δρ_min_ = −0.87 e Å^−^^3^

### Special details

**Table:**

Experimental. The crystal was placed in the cold stream of an Oxford Cyrosystems Cobra open-flow nitrogen cryostat (Cosier & Glazer, 1986) operating at 100.0 (1) K.
Geometry. All e.s.d.'s (except the e.s.d. in the dihedral angle between two l.s. planes) are estimated using the full covariance matrix. The cell e.s.d.'s are taken into account individually in the estimation of e.s.d.'s in distances, angles and torsion angles; correlations between e.s.d.'s in cell parameters are only used when they are defined by crystal symmetry. An approximate (isotropic) treatment of cell e.s.d.'s is used for estimating e.s.d.'s involving l.s. planes.
Refinement. Refinement of *F*^2^ against ALL reflections. The weighted *R*-factor *wR* and goodness of fit *S* are based on *F*^2^, conventional *R*-factors *R* are based on *F*, with *F* set to zero for negative *F*^2^. The threshold expression of *F*^2^ > σ(*F*^2^) is used only for calculating *R*-factors(gt) *etc*. and is not relevant to the choice of reflections for refinement. *R*-factors based on *F*^2^ are statistically about twice as large as those based on *F*, and *R*- factors based on ALL data will be even larger.

### Fractional atomic coordinates and isotropic or equivalent isotropic displacement parameters (Å^2^)

**Table:**

	*x*	*y*	*z*	*U*_iso_*/*U*_eq_	
Mn1A	0.31388 (4)	0.14087 (2)	0.51808 (2)	0.01188 (10)	
Cl1A	0.18695 (6)	0.14030 (4)	0.42229 (4)	0.01801 (14)	
O1A	0.25814 (18)	0.21411 (11)	0.59235 (10)	0.0164 (4)	
O2A	0.47129 (17)	0.22959 (11)	0.52533 (10)	0.0146 (4)	
N1A	0.1949 (2)	0.03602 (13)	0.53648 (12)	0.0132 (5)	
N2A	0.3938 (2)	0.05414 (13)	0.46243 (11)	0.0118 (4)	
C1A	0.1616 (2)	0.19866 (17)	0.63162 (14)	0.0135 (5)	
C2A	0.1319 (3)	0.26952 (17)	0.67985 (15)	0.0154 (5)	
C3A	0.0328 (3)	0.24975 (18)	0.72168 (15)	0.0175 (6)	
H3A	0.0137	0.2962	0.7532	0.021*	
C4A	−0.0414 (3)	0.16473 (18)	0.72024 (14)	0.0158 (6)	
C5A	−0.0135 (3)	0.09720 (18)	0.67274 (14)	0.0158 (6)	
H5A	−0.0608	0.0402	0.6697	0.019*	
C6A	0.0862 (3)	0.11232 (17)	0.62797 (14)	0.0135 (5)	
C7A	0.1090 (2)	0.03709 (17)	0.58203 (14)	0.0151 (5)	
H7A	0.0577	−0.0167	0.5847	0.018*	
C8A	0.2104 (2)	−0.04378 (16)	0.49332 (14)	0.0131 (5)	
C9A	0.1259 (2)	−0.12690 (17)	0.48770 (14)	0.0153 (5)	
H9A	0.0521	−0.1334	0.5120	0.018*	
C10A	0.1536 (3)	−0.19873 (17)	0.44564 (15)	0.0174 (6)	
H10A	0.0984	−0.2542	0.4420	0.021*	
C11A	0.2627 (3)	−0.18983 (17)	0.40853 (15)	0.0173 (6)	
H11A	0.2799	−0.2393	0.3804	0.021*	
C12A	0.3460 (3)	−0.10768 (17)	0.41325 (15)	0.0159 (6)	
H12A	0.4196	−0.1018	0.3888	0.019*	
C13A	0.3184 (2)	−0.03402 (16)	0.45504 (13)	0.0118 (5)	
C14A	0.4975 (2)	0.07412 (16)	0.42955 (14)	0.0126 (5)	
H14A	0.5198	0.0276	0.4000	0.015*	
C15A	0.5796 (2)	0.15909 (16)	0.43410 (14)	0.0130 (5)	
C16A	0.6860 (2)	0.16599 (17)	0.39256 (14)	0.0131 (5)	
H16A	0.6957	0.1155	0.3616	0.016*	
C17A	0.7752 (3)	0.24511 (17)	0.39671 (14)	0.0143 (5)	
C18A	0.7531 (3)	0.31927 (16)	0.44348 (14)	0.0146 (5)	
H18A	0.8103	0.3736	0.4454	0.018*	
C19A	0.6538 (3)	0.31784 (17)	0.48650 (15)	0.0144 (5)	
C20A	0.5635 (2)	0.23477 (16)	0.48243 (14)	0.0126 (5)	
C21A	0.2087 (3)	0.36459 (17)	0.68473 (16)	0.0199 (6)	
C22A	0.1602 (3)	0.42991 (19)	0.74011 (19)	0.0332 (8)	
H22A	0.1746	0.4238	0.7849	0.050*	
H22B	0.0685	0.4186	0.7293	0.050*	
H22C	0.2072	0.4882	0.7408	0.050*	
C23A	0.3546 (3)	0.38322 (18)	0.70484 (16)	0.0235 (6)	
H23A	0.3881	0.3444	0.6700	0.035*	
H23B	0.3654	0.3744	0.7489	0.035*	
H23C	0.4015	0.4425	0.7081	0.035*	
C24A	0.1903 (3)	0.38074 (18)	0.61482 (17)	0.0240 (6)	
H24A	0.2134	0.3379	0.5782	0.036*	
H24B	0.2455	0.4380	0.6171	0.036*	
H24C	0.1007	0.3764	0.6056	0.036*	
C25A	−0.1421 (3)	0.14974 (19)	0.77303 (15)	0.0195 (6)	
C26A	−0.0728 (3)	0.1542 (3)	0.83987 (17)	0.0341 (4)	
H26A	−0.0281	0.1111	0.8301	0.051*	
H26B	−0.1357	0.1430	0.8722	0.051*	
H26C	−0.0107	0.2112	0.8596	0.051*	
C27A	−0.2465 (3)	0.0606 (2)	0.7452 (2)	0.0350 (8)	
H27A	−0.2066	0.0155	0.7392	0.053*	
H27B	−0.2874	0.0553	0.7012	0.053*	
H27C	−0.3111	0.0551	0.7776	0.053*	
C28A	−0.2122 (3)	0.2194 (2)	0.78932 (19)	0.0300 (7)	
H28A	−0.1500	0.2758	0.8112	0.045*	
H28B	−0.2779	0.2068	0.8201	0.045*	
H28C	−0.2527	0.2190	0.7469	0.045*	
C29A	0.6423 (3)	0.40175 (17)	0.53913 (15)	0.0171 (6)	
C30A	0.7391 (3)	0.48344 (18)	0.52845 (19)	0.0313 (8)	
H30A	0.7257	0.5347	0.5599	0.047*	
H30B	0.7251	0.4829	0.4814	0.047*	
H30C	0.8269	0.4838	0.5373	0.047*	
C31A	0.5041 (3)	0.40935 (17)	0.53201 (15)	0.0194 (6)	
H31A	0.4993	0.4615	0.5663	0.029*	
H31B	0.4410	0.3595	0.5390	0.029*	
H31C	0.4858	0.4118	0.4863	0.029*	
C32A	0.6727 (3)	0.40166 (19)	0.61313 (16)	0.0235 (7)	
H32A	0.6656	0.4540	0.6461	0.035*	
H32B	0.7599	0.3990	0.6184	0.035*	
H32C	0.6116	0.3519	0.6213	0.035*	
C33A	0.8967 (3)	0.25655 (17)	0.35688 (15)	0.0168 (6)	
C34A	0.8929 (3)	0.3135 (2)	0.30992 (17)	0.0341 (4)	
H34A	0.8874	0.3690	0.3381	0.051*	
H34B	0.8180	0.2848	0.2762	0.051*	
H34C	0.9710	0.3221	0.2864	0.051*	
C35A	0.9076 (3)	0.16952 (18)	0.31127 (16)	0.0210 (6)	
H35A	0.9085	0.1330	0.3395	0.031*	
H35B	0.9870	0.1794	0.2890	0.031*	
H35C	0.8342	0.1414	0.2765	0.031*	
C36A	1.0181 (3)	0.30190 (18)	0.40908 (16)	0.0225 (6)	
H36A	1.0228	0.2660	0.4373	0.034*	
H36B	1.0131	0.3571	0.4382	0.034*	
H36C	1.0947	0.3112	0.3845	0.034*	
Mn1B	0.81404 (4)	0.14792 (2)	0.05624 (2)	0.01257 (10)	
Cl1B	0.67345 (6)	0.13879 (4)	0.14576 (4)	0.01833 (14)	
O1B	0.96506 (17)	0.24070 (11)	0.09672 (10)	0.0159 (4)	
O2B	0.75671 (18)	0.21918 (12)	0.01644 (10)	0.0179 (4)	
N1B	0.8995 (2)	0.06442 (14)	0.07023 (12)	0.0134 (5)	
N2B	0.7023 (2)	0.04165 (13)	−0.01512 (12)	0.0137 (5)	
C1B	1.0666 (3)	0.24648 (17)	0.13647 (14)	0.0137 (5)	
C2B	1.1640 (3)	0.32922 (17)	0.16545 (14)	0.0149 (6)	
C3B	1.2674 (3)	0.33187 (17)	0.20758 (14)	0.0160 (6)	
H3B	1.3296	0.3859	0.2281	0.019*	
C4B	1.2860 (3)	0.25854 (17)	0.22202 (14)	0.0145 (5)	
C5B	1.1941 (3)	0.18003 (17)	0.19211 (14)	0.0148 (5)	
H5B	1.2032	0.1306	0.2002	0.018*	
C6B	1.0843 (2)	0.17193 (17)	0.14869 (14)	0.0135 (5)	
C7B	1.0020 (3)	0.08618 (17)	0.11369 (14)	0.0146 (5)	
H7B	1.0235	0.0408	0.1228	0.018*	
C8B	0.8300 (2)	−0.02452 (16)	0.03402 (14)	0.0139 (5)	
C9B	0.8616 (3)	−0.09657 (17)	0.03990 (15)	0.0160 (6)	
H9B	0.9347	−0.0886	0.0686	0.019*	
C10B	0.7843 (3)	−0.17947 (17)	0.00308 (14)	0.0169 (6)	
H10B	0.8062	−0.2274	0.0066	0.020*	
C11B	0.6740 (3)	−0.19231 (17)	−0.03922 (15)	0.0170 (6)	
H11B	0.6207	−0.2486	−0.0625	0.020*	
C12B	0.6431 (3)	−0.12087 (17)	−0.04682 (14)	0.0161 (6)	
H12B	0.5700	−0.1293	−0.0757	0.019*	
C13B	0.7222 (3)	−0.03696 (17)	−0.01096 (14)	0.0148 (5)	
C14B	0.6164 (2)	0.04058 (17)	−0.06111 (14)	0.0135 (5)	
H14B	0.5681	−0.0137	−0.0911	0.016*	
C15B	0.5893 (3)	0.11481 (17)	−0.06973 (14)	0.0146 (5)	
C16B	0.4907 (3)	0.09798 (17)	−0.12289 (14)	0.0156 (5)	
H16B	0.4466	0.0406	−0.1490	0.019*	
C17B	0.4587 (3)	0.16487 (18)	−0.13671 (14)	0.0161 (6)	
C18B	0.5239 (3)	0.25035 (17)	−0.09345 (14)	0.0157 (6)	
H18B	0.5009	0.2958	−0.1015	0.019*	
C19B	0.6198 (3)	0.27152 (17)	−0.03982 (15)	0.0147 (5)	
C20B	0.6577 (2)	0.20141 (17)	−0.02949 (14)	0.0149 (5)	
C21B	1.1547 (3)	0.41047 (17)	0.14861 (15)	0.0170 (6)	
C22B	1.2720 (3)	0.49113 (17)	0.18278 (16)	0.0246 (7)	
H22D	1.3508	0.4805	0.1659	0.037*	
H22E	1.2770	0.5032	0.2323	0.037*	
H22F	1.2619	0.5403	0.1715	0.037*	
C23B	1.0298 (3)	0.43111 (19)	0.17463 (17)	0.0238 (6)	
H23D	1.0337	0.4440	0.2243	0.036*	
H23E	0.9548	0.3816	0.1533	0.036*	
H23F	1.0232	0.4806	0.1625	0.036*	
C24B	1.1539 (3)	0.39474 (17)	0.07001 (15)	0.0186 (6)	
H24D	1.2350	0.3863	0.0550	0.028*	
H24E	1.1436	0.4443	0.0595	0.028*	
H24F	1.0827	0.3437	0.0463	0.028*	
C25B	1.4080 (3)	0.27108 (17)	0.26781 (15)	0.0162 (6)	
C26B	1.4039 (3)	0.3293 (2)	0.34077 (17)	0.0341 (4)	
H26D	1.3305	0.3007	0.3612	0.051*	
H26E	1.3954	0.3834	0.3385	0.051*	
H26F	1.4832	0.3406	0.3687	0.051*	
C27B	1.5314 (3)	0.31503 (19)	0.23714 (16)	0.0218 (6)	
H27D	1.5355	0.2780	0.1914	0.033*	
H27E	1.6078	0.3244	0.2665	0.033*	
H27F	1.5275	0.3700	0.2345	0.033*	
C28B	1.4175 (3)	0.18426 (18)	0.27298 (16)	0.0197 (6)	
H28D	1.4199	0.1464	0.2273	0.030*	
H28E	1.3427	0.1572	0.2934	0.030*	
H28F	1.4957	0.1949	0.3015	0.030*	
C29B	0.6846 (3)	0.36594 (17)	0.00566 (16)	0.0170 (6)	
C30B	0.6189 (3)	0.42843 (18)	−0.01036 (17)	0.0229 (6)	
H30D	0.5277	0.4100	−0.0038	0.034*	
H30E	0.6284	0.4278	−0.0577	0.034*	
H30F	0.6595	0.4865	0.0204	0.034*	
C31B	0.8295 (3)	0.39528 (18)	−0.00812 (17)	0.0241 (7)	
H31D	0.8718	0.3561	0.0003	0.036*	
H31E	0.8719	0.4532	0.0224	0.036*	
H31F	0.8351	0.3948	−0.0556	0.036*	
C32B	0.6733 (3)	0.37385 (18)	0.08280 (16)	0.0227 (6)	
H32D	0.5829	0.3605	0.0915	0.034*	
H32E	0.7201	0.4323	0.1109	0.034*	
H32F	0.7097	0.3337	0.0943	0.034*	
C33B	0.3620 (3)	0.15007 (19)	−0.19793 (15)	0.0201 (6)	
C34B	0.4377 (3)	0.1670 (3)	−0.25857 (17)	0.0341 (4)	
H34D	0.4892	0.1282	−0.2718	0.051*	
H34E	0.4942	0.2263	−0.2448	0.051*	
H34F	0.3775	0.1573	−0.2973	0.051*	
C35B	0.2673 (4)	0.0572 (2)	−0.2201 (2)	0.0399 (9)	
H35D	0.3149	0.0165	−0.2367	0.060*	
H35E	0.2042	0.0506	−0.2565	0.060*	
H35F	0.2231	0.0462	−0.1810	0.060*	
C36B	0.2804 (3)	0.2125 (2)	−0.17889 (17)	0.0254 (7)	
H36D	0.2182	0.2000	−0.2175	0.038*	
H36E	0.3368	0.2716	−0.1679	0.038*	
H36F	0.2349	0.2051	−0.1393	0.038*	

### Atomic displacement parameters (Å^2^)

**Table:**

	*U*^11^	*U*^22^	*U*^33^	*U*^12^	*U*^13^	*U*^23^
Mn1A	0.01029 (19)	0.00976 (18)	0.0144 (2)	0.00330 (14)	0.00419 (16)	0.00183 (16)
Cl1A	0.0143 (3)	0.0197 (3)	0.0216 (4)	0.0054 (2)	0.0015 (3)	0.0087 (3)
O1A	0.0166 (9)	0.0130 (9)	0.0185 (10)	0.0051 (7)	0.0093 (8)	0.0029 (8)
O2A	0.0113 (9)	0.0124 (9)	0.0164 (10)	0.0021 (7)	0.0060 (8)	0.0008 (7)
N1A	0.0113 (10)	0.0128 (10)	0.0155 (12)	0.0046 (8)	0.0021 (9)	0.0038 (9)
N2A	0.0114 (10)	0.0098 (10)	0.0133 (11)	0.0038 (8)	0.0014 (9)	0.0018 (8)
C1A	0.0128 (13)	0.0166 (13)	0.0113 (13)	0.0058 (10)	0.0034 (10)	0.0036 (10)
C2A	0.0136 (12)	0.0167 (13)	0.0145 (13)	0.0066 (10)	0.0011 (11)	0.0012 (11)
C3A	0.0135 (13)	0.0246 (14)	0.0157 (14)	0.0103 (11)	0.0022 (11)	0.0039 (11)
C4A	0.0107 (12)	0.0253 (14)	0.0139 (14)	0.0081 (11)	0.0037 (11)	0.0073 (11)
C5A	0.0119 (12)	0.0198 (13)	0.0147 (14)	0.0037 (10)	0.0008 (11)	0.0052 (11)
C6A	0.0123 (12)	0.0158 (13)	0.0137 (13)	0.0061 (10)	0.0024 (11)	0.0048 (11)
C7A	0.0111 (12)	0.0177 (13)	0.0169 (14)	0.0046 (10)	0.0014 (11)	0.0058 (11)
C8A	0.0115 (12)	0.0146 (12)	0.0130 (13)	0.0039 (10)	−0.0009 (10)	0.0039 (10)
C9A	0.0087 (12)	0.0190 (13)	0.0166 (14)	0.0013 (10)	0.0009 (11)	0.0064 (11)
C10A	0.0182 (14)	0.0131 (12)	0.0211 (15)	0.0026 (10)	−0.0015 (11)	0.0078 (11)
C11A	0.0207 (14)	0.0111 (12)	0.0178 (14)	0.0066 (10)	−0.0014 (11)	−0.0005 (10)
C12A	0.0135 (13)	0.0152 (13)	0.0183 (14)	0.0047 (10)	0.0009 (11)	0.0043 (11)
C13A	0.0112 (12)	0.0117 (12)	0.0113 (13)	0.0033 (9)	0.0008 (10)	0.0023 (10)
C14A	0.0083 (12)	0.0124 (12)	0.0150 (13)	0.0044 (9)	0.0010 (10)	0.0001 (10)
C15A	0.0106 (12)	0.0108 (12)	0.0154 (13)	0.0031 (10)	−0.0001 (10)	0.0013 (10)
C16A	0.0113 (12)	0.0160 (12)	0.0116 (13)	0.0057 (10)	0.0024 (10)	0.0025 (10)
C17A	0.0116 (12)	0.0173 (13)	0.0146 (13)	0.0050 (10)	0.0031 (10)	0.0054 (10)
C18A	0.0137 (12)	0.0110 (12)	0.0169 (13)	−0.0005 (10)	0.0016 (11)	0.0054 (10)
C19A	0.0124 (12)	0.0135 (12)	0.0166 (14)	0.0045 (10)	0.0021 (11)	0.0032 (11)
C20A	0.0106 (12)	0.0146 (12)	0.0137 (13)	0.0056 (10)	0.0009 (10)	0.0043 (10)
C21A	0.0226 (15)	0.0139 (13)	0.0216 (15)	0.0076 (11)	0.0082 (12)	0.0014 (11)
C22A	0.0351 (18)	0.0195 (15)	0.039 (2)	0.0091 (13)	0.0176 (16)	−0.0001 (14)
C23A	0.0228 (15)	0.0180 (14)	0.0227 (16)	0.0022 (11)	0.0012 (13)	0.0002 (12)
C24A	0.0231 (15)	0.0192 (14)	0.0295 (17)	0.0058 (12)	0.0025 (13)	0.0082 (13)
C25A	0.0124 (13)	0.0319 (15)	0.0176 (15)	0.0092 (11)	0.0075 (12)	0.0104 (13)
C26A	0.0249 (8)	0.0606 (11)	0.0232 (8)	0.0190 (8)	0.0054 (8)	0.0165 (9)
C27A	0.0336 (18)	0.0309 (17)	0.043 (2)	0.0093 (14)	0.0251 (16)	0.0162 (16)
C28A	0.0257 (16)	0.0282 (16)	0.0359 (19)	0.0105 (13)	0.0148 (15)	0.0076 (15)
C29A	0.0164 (13)	0.0121 (12)	0.0199 (14)	0.0022 (10)	0.0023 (11)	0.0031 (11)
C30A	0.0295 (17)	0.0122 (13)	0.047 (2)	0.0046 (12)	0.0157 (15)	0.0042 (14)
C31A	0.0235 (15)	0.0129 (13)	0.0221 (15)	0.0089 (11)	0.0046 (12)	0.0031 (11)
C32A	0.0228 (15)	0.0215 (14)	0.0234 (16)	0.0115 (12)	−0.0036 (13)	−0.0016 (12)
C33A	0.0141 (13)	0.0183 (13)	0.0194 (14)	0.0056 (10)	0.0069 (11)	0.0074 (11)
C34A	0.0249 (8)	0.0606 (11)	0.0232 (8)	0.0190 (8)	0.0054 (8)	0.0165 (9)
C35A	0.0173 (14)	0.0215 (14)	0.0202 (15)	0.0059 (11)	0.0088 (12)	0.0010 (12)
C36A	0.0153 (14)	0.0200 (14)	0.0279 (16)	0.0038 (11)	0.0029 (12)	0.0029 (12)
Mn1B	0.01071 (19)	0.01110 (19)	0.0154 (2)	0.00394 (15)	−0.00031 (17)	0.00292 (16)
Cl1B	0.0148 (3)	0.0195 (3)	0.0186 (3)	0.0058 (2)	0.0037 (3)	0.0023 (3)
O1B	0.0118 (9)	0.0131 (9)	0.0221 (10)	0.0036 (7)	−0.0008 (8)	0.0047 (8)
O2B	0.0177 (10)	0.0150 (9)	0.0204 (11)	0.0049 (8)	−0.0052 (8)	0.0048 (8)
N1B	0.0120 (11)	0.0129 (10)	0.0149 (11)	0.0042 (8)	0.0023 (9)	0.0036 (9)
N2B	0.0121 (10)	0.0129 (10)	0.0168 (12)	0.0054 (8)	0.0034 (9)	0.0041 (9)
C1B	0.0136 (13)	0.0147 (12)	0.0131 (13)	0.0069 (10)	0.0023 (11)	0.0023 (10)
C2B	0.0154 (13)	0.0146 (13)	0.0146 (13)	0.0073 (10)	0.0027 (11)	0.0021 (11)
C3B	0.0152 (13)	0.0148 (12)	0.0156 (14)	0.0040 (10)	0.0029 (11)	0.0020 (10)
C4B	0.0136 (12)	0.0185 (13)	0.0118 (13)	0.0067 (10)	0.0027 (10)	0.0038 (10)
C5B	0.0156 (13)	0.0181 (13)	0.0149 (14)	0.0092 (10)	0.0055 (11)	0.0071 (11)
C6B	0.0094 (12)	0.0165 (13)	0.0145 (13)	0.0029 (10)	0.0037 (10)	0.0056 (11)
C7B	0.0147 (13)	0.0156 (13)	0.0171 (14)	0.0074 (10)	0.0066 (11)	0.0075 (11)
C8B	0.0118 (12)	0.0145 (12)	0.0137 (13)	0.0035 (10)	0.0033 (10)	0.0025 (10)
C9B	0.0166 (13)	0.0146 (13)	0.0172 (14)	0.0066 (10)	0.0010 (11)	0.0036 (11)
C10B	0.0212 (14)	0.0144 (13)	0.0172 (14)	0.0085 (11)	0.0049 (11)	0.0053 (11)
C11B	0.0178 (13)	0.0142 (12)	0.0182 (14)	0.0048 (10)	0.0063 (11)	0.0040 (11)
C12B	0.0136 (13)	0.0173 (13)	0.0135 (13)	0.0041 (10)	0.0040 (11)	−0.0004 (11)
C13B	0.0137 (13)	0.0157 (12)	0.0167 (14)	0.0064 (10)	0.0058 (11)	0.0056 (10)
C14B	0.0134 (12)	0.0150 (12)	0.0107 (13)	0.0049 (10)	0.0025 (10)	0.0016 (10)
C15B	0.0131 (13)	0.0180 (13)	0.0117 (13)	0.0047 (10)	0.0016 (11)	0.0036 (11)
C16B	0.0134 (13)	0.0172 (13)	0.0135 (13)	0.0051 (10)	0.0026 (11)	0.0008 (11)
C17B	0.0136 (13)	0.0237 (14)	0.0128 (13)	0.0095 (11)	0.0045 (11)	0.0047 (11)
C18B	0.0155 (13)	0.0190 (13)	0.0176 (14)	0.0106 (11)	0.0060 (11)	0.0080 (11)
C19B	0.0138 (13)	0.0164 (13)	0.0152 (14)	0.0064 (10)	0.0033 (11)	0.0053 (11)
C20B	0.0101 (12)	0.0208 (13)	0.0142 (14)	0.0054 (10)	0.0028 (11)	0.0058 (11)
C21B	0.0199 (14)	0.0118 (12)	0.0205 (14)	0.0065 (10)	0.0002 (11)	0.0052 (11)
C22B	0.0305 (16)	0.0140 (13)	0.0251 (16)	0.0037 (12)	−0.0059 (13)	0.0032 (12)
C23B	0.0282 (16)	0.0189 (14)	0.0274 (16)	0.0134 (12)	0.0059 (13)	0.0055 (12)
C24B	0.0204 (14)	0.0139 (13)	0.0217 (15)	0.0043 (10)	0.0007 (12)	0.0069 (11)
C25B	0.0133 (13)	0.0185 (13)	0.0168 (14)	0.0068 (10)	−0.0014 (11)	0.0037 (11)
C26B	0.0249 (8)	0.0606 (11)	0.0232 (8)	0.0190 (8)	0.0054 (8)	0.0165 (9)
C27B	0.0138 (13)	0.0247 (15)	0.0269 (16)	0.0054 (11)	0.0007 (12)	0.0088 (12)
C28B	0.0138 (13)	0.0227 (14)	0.0219 (15)	0.0066 (11)	−0.0025 (12)	0.0050 (12)
C29B	0.0151 (13)	0.0150 (13)	0.0222 (15)	0.0061 (10)	−0.0005 (11)	0.0062 (11)
C30B	0.0221 (15)	0.0182 (14)	0.0290 (17)	0.0062 (11)	−0.0012 (13)	0.0083 (12)
C31B	0.0194 (14)	0.0200 (14)	0.0332 (18)	0.0042 (11)	−0.0012 (13)	0.0107 (13)
C32B	0.0262 (15)	0.0181 (14)	0.0223 (16)	0.0078 (12)	0.0004 (13)	0.0032 (12)
C33B	0.0164 (14)	0.0296 (15)	0.0172 (15)	0.0117 (12)	0.0001 (12)	0.0068 (12)
C34B	0.0249 (8)	0.0606 (11)	0.0232 (8)	0.0190 (8)	0.0054 (8)	0.0165 (9)
C35B	0.041 (2)	0.0268 (17)	0.045 (2)	0.0068 (15)	−0.0263 (18)	0.0041 (16)
C36B	0.0183 (14)	0.0331 (16)	0.0287 (17)	0.0125 (13)	−0.0041 (13)	0.0108 (14)

### Geometric parameters (Å, °)

**Table:**

Mn1A—O1A	1.8660 (19)	Mn1B—O1B	1.8682 (18)
Mn1A—O2A	1.8706 (18)	Mn1B—O2B	1.8710 (19)
Mn1A—N2A	1.980 (2)	Mn1B—N2B	1.981 (2)
Mn1A—N1A	1.991 (2)	Mn1B—N1B	1.982 (2)
Mn1A—Cl1A	2.3671 (8)	Mn1B—Cl1B	2.3714 (8)
O1A—C1A	1.320 (3)	O1B—C1B	1.319 (3)
O2A—C20A	1.319 (3)	O2B—C20B	1.316 (3)
N1A—C7A	1.305 (4)	N1B—C7B	1.300 (3)
N1A—C8A	1.429 (3)	N1B—C8B	1.425 (3)
N2A—C14A	1.309 (3)	N2B—C14B	1.305 (3)
N2A—C13A	1.431 (3)	N2B—C13B	1.433 (3)
C1A—C2A	1.421 (4)	C1B—C6B	1.419 (4)
C1A—C6A	1.423 (4)	C1B—C2B	1.427 (4)
C2A—C3A	1.387 (4)	C2B—C3B	1.383 (4)
C2A—C21A	1.546 (4)	C2B—C21B	1.540 (4)
C3A—C4A	1.413 (4)	C3B—C4B	1.423 (4)
C3A—H3A	0.9300	C3B—H3B	0.9300
C4A—C5A	1.366 (4)	C4B—C5B	1.358 (4)
C4A—C25A	1.542 (4)	C4B—C25B	1.532 (4)
C5A—C6A	1.419 (4)	C5B—C6B	1.421 (4)
C5A—H5A	0.9300	C5B—H5B	0.9300
C6A—C7A	1.422 (4)	C6B—C7B	1.418 (4)
C7A—H7A	0.9300	C7B—H7B	0.9300
C8A—C13A	1.393 (4)	C8B—C9B	1.395 (4)
C8A—C9A	1.400 (3)	C8B—C13B	1.400 (4)
C9A—C10A	1.373 (4)	C9B—C10B	1.376 (4)
C9A—H9A	0.9300	C9B—H9B	0.9300
C10A—C11A	1.388 (4)	C10B—C11B	1.387 (4)
C10A—H10A	0.9300	C10B—H10B	0.9300
C11A—C12A	1.387 (4)	C11B—C12B	1.393 (4)
C11A—H11A	0.9300	C11B—H11B	0.9300
C12A—C13A	1.395 (4)	C12B—C13B	1.389 (4)
C12A—H12A	0.9300	C12B—H12B	0.9300
C14A—C15A	1.421 (3)	C14B—C15B	1.427 (4)
C14A—H14A	0.9300	C14B—H14B	0.9300
C15A—C20A	1.417 (3)	C15B—C20B	1.412 (4)
C15A—C16A	1.418 (4)	C15B—C16B	1.415 (4)
C16A—C17A	1.373 (4)	C16B—C17B	1.376 (4)
C16A—H16A	0.9300	C16B—H16B	0.9300
C17A—C18A	1.411 (4)	C17B—C18B	1.410 (4)
C17A—C33A	1.531 (4)	C17B—C33B	1.530 (4)
C18A—C19A	1.378 (4)	C18B—C19B	1.385 (4)
C18A—H18A	0.9300	C18B—H18B	0.9300
C19A—C20A	1.429 (4)	C19B—C20B	1.431 (4)
C19A—C29A	1.537 (4)	C19B—C29B	1.530 (4)
C21A—C22A	1.533 (4)	C21B—C22B	1.531 (4)
C21A—C23A	1.537 (4)	C21B—C24B	1.532 (4)
C21A—C24A	1.541 (4)	C21B—C23B	1.542 (4)
C22A—H22A	0.9600	C22B—H22D	0.9600
C22A—H22B	0.9600	C22B—H22E	0.9600
C22A—H22C	0.9600	C22B—H22F	0.9600
C23A—H23A	0.9600	C23B—H23D	0.9600
C23A—H23B	0.9600	C23B—H23E	0.9600
C23A—H23C	0.9600	C23B—H23F	0.9600
C24A—H24A	0.9600	C24B—H24D	0.9600
C24A—H24B	0.9600	C24B—H24E	0.9600
C24A—H24C	0.9600	C24B—H24F	0.9600
C25A—C26A	1.515 (4)	C25B—C26B	1.519 (4)
C25A—C27A	1.529 (4)	C25B—C28B	1.535 (4)
C25A—C28A	1.536 (4)	C25B—C27B	1.546 (4)
C26A—H26A	0.9600	C26B—H26D	0.9600
C26A—H26B	0.9600	C26B—H26E	0.9600
C26A—H26C	0.9600	C26B—H26F	0.9600
C27A—H27A	0.9600	C27B—H27D	0.9600
C27A—H27B	0.9600	C27B—H27E	0.9600
C27A—H27C	0.9600	C27B—H27F	0.9600
C28A—H28A	0.9600	C28B—H28D	0.9600
C28A—H28B	0.9600	C28B—H28E	0.9600
C28A—H28C	0.9600	C28B—H28F	0.9600
C29A—C32A	1.533 (4)	C29B—C32B	1.534 (4)
C29A—C30A	1.537 (4)	C29B—C30B	1.539 (4)
C29A—C31A	1.542 (4)	C29B—C31B	1.543 (4)
C30A—H30A	0.9600	C30B—H30D	0.9600
C30A—H30B	0.9600	C30B—H30E	0.9600
C30A—H30C	0.9600	C30B—H30F	0.9600
C31A—H31A	0.9600	C31B—H31D	0.9600
C31A—H31B	0.9600	C31B—H31E	0.9600
C31A—H31C	0.9600	C31B—H31F	0.9600
C32A—H32A	0.9600	C32B—H32D	0.9600
C32A—H32B	0.9600	C32B—H32E	0.9600
C32A—H32C	0.9600	C32B—H32F	0.9600
C33A—C35A	1.523 (4)	C33B—C34B	1.524 (4)
C33A—C36A	1.527 (4)	C33B—C35B	1.527 (4)
C33A—C34A	1.550 (4)	C33B—C36B	1.531 (4)
C34A—H34A	0.9600	C34B—H34D	0.9600
C34A—H34B	0.9600	C34B—H34E	0.9600
C34A—H34C	0.9600	C34B—H34F	0.9600
C35A—H35A	0.9600	C35B—H35D	0.9600
C35A—H35B	0.9600	C35B—H35E	0.9600
C35A—H35C	0.9600	C35B—H35F	0.9600
C36A—H36A	0.9600	C36B—H36D	0.9600
C36A—H36B	0.9600	C36B—H36E	0.9600
C36A—H36C	0.9600	C36B—H36F	0.9600
			
O1A—Mn1A—O2A	90.39 (8)	O1B—Mn1B—O2B	89.77 (8)
O1A—Mn1A—N2A	162.68 (9)	O1B—Mn1B—N2B	157.14 (9)
O2A—Mn1A—N2A	89.72 (8)	O2B—Mn1B—N2B	91.08 (8)
O1A—Mn1A—N1A	90.80 (9)	O1B—Mn1B—N1B	90.30 (8)
O2A—Mn1A—N1A	155.86 (9)	O2B—Mn1B—N1B	161.87 (9)
N2A—Mn1A—N1A	82.16 (9)	N2B—Mn1B—N1B	81.95 (9)
O1A—Mn1A—Cl1A	101.53 (6)	O1B—Mn1B—Cl1B	106.37 (7)
O2A—Mn1A—Cl1A	104.82 (6)	O2B—Mn1B—Cl1B	101.50 (7)
N2A—Mn1A—Cl1A	95.17 (7)	N2B—Mn1B—Cl1B	95.83 (7)
N1A—Mn1A—Cl1A	98.55 (7)	N1B—Mn1B—Cl1B	95.87 (7)
C1A—O1A—Mn1A	132.19 (17)	C1B—O1B—Mn1B	131.46 (16)
C20A—O2A—Mn1A	129.94 (16)	C20B—O2B—Mn1B	131.52 (17)
C7A—N1A—C8A	121.7 (2)	C7B—N1B—C8B	121.7 (2)
C7A—N1A—Mn1A	125.27 (18)	C7B—N1B—Mn1B	124.13 (18)
C8A—N1A—Mn1A	113.01 (17)	C8B—N1B—Mn1B	113.81 (16)
C14A—N2A—C13A	122.8 (2)	C14B—N2B—C13B	121.8 (2)
C14A—N2A—Mn1A	123.68 (17)	C14B—N2B—Mn1B	124.98 (17)
C13A—N2A—Mn1A	113.18 (16)	C13B—N2B—Mn1B	113.23 (17)
O1A—C1A—C2A	119.8 (2)	O1B—C1B—C6B	121.7 (2)
O1A—C1A—C6A	121.9 (2)	O1B—C1B—C2B	119.1 (2)
C2A—C1A—C6A	118.3 (2)	C6B—C1B—C2B	119.1 (2)
C3A—C2A—C1A	117.5 (2)	C3B—C2B—C1B	116.8 (2)
C3A—C2A—C21A	122.1 (2)	C3B—C2B—C21B	122.3 (2)
C1A—C2A—C21A	120.4 (2)	C1B—C2B—C21B	120.9 (2)
C2A—C3A—C4A	125.3 (3)	C2B—C3B—C4B	125.2 (2)
C2A—C3A—H3A	117.3	C2B—C3B—H3B	117.4
C4A—C3A—H3A	117.3	C4B—C3B—H3B	117.4
C5A—C4A—C3A	116.6 (3)	C5B—C4B—C3B	116.9 (2)
C5A—C4A—C25A	122.0 (3)	C5B—C4B—C25B	123.7 (2)
C3A—C4A—C25A	121.3 (2)	C3B—C4B—C25B	119.3 (2)
C4A—C5A—C6A	121.3 (3)	C4B—C5B—C6B	121.3 (2)
C4A—C5A—H5A	119.3	C4B—C5B—H5B	119.3
C6A—C5A—H5A	119.3	C6B—C5B—H5B	119.3
C5A—C6A—C7A	116.4 (2)	C7B—C6B—C1B	122.2 (2)
C5A—C6A—C1A	121.0 (2)	C7B—C6B—C5B	117.0 (2)
C7A—C6A—C1A	122.6 (3)	C1B—C6B—C5B	120.6 (2)
N1A—C7A—C6A	126.5 (3)	N1B—C7B—C6B	126.7 (2)
N1A—C7A—H7A	116.8	N1B—C7B—H7B	116.6
C6A—C7A—H7A	116.8	C6B—C7B—H7B	116.6
C13A—C8A—C9A	120.5 (2)	C9B—C8B—C13B	119.8 (2)
C13A—C8A—N1A	114.9 (2)	C9B—C8B—N1B	125.6 (2)
C9A—C8A—N1A	124.5 (2)	C13B—C8B—N1B	114.6 (2)
C10A—C9A—C8A	118.9 (3)	C10B—C9B—C8B	119.8 (2)
C10A—C9A—H9A	120.6	C10B—C9B—H9B	120.1
C8A—C9A—H9A	120.6	C8B—C9B—H9B	120.1
C9A—C10A—C11A	121.1 (2)	C9B—C10B—C11B	120.7 (2)
C9A—C10A—H10A	119.5	C9B—C10B—H10B	119.6
C11A—C10A—H10A	119.5	C11B—C10B—H10B	119.6
C12A—C11A—C10A	120.4 (2)	C10B—C11B—C12B	120.0 (2)
C12A—C11A—H11A	119.8	C10B—C11B—H11B	120.0
C10A—C11A—H11A	119.8	C12B—C11B—H11B	120.0
C11A—C12A—C13A	119.3 (3)	C13B—C12B—C11B	119.6 (3)
C11A—C12A—H12A	120.3	C13B—C12B—H12B	120.2
C13A—C12A—H12A	120.3	C11B—C12B—H12B	120.2
C8A—C13A—C12A	119.8 (2)	C12B—C13B—C8B	120.0 (2)
C8A—C13A—N2A	115.4 (2)	C12B—C13B—N2B	125.1 (2)
C12A—C13A—N2A	124.8 (2)	C8B—C13B—N2B	114.9 (2)
N2A—C14A—C15A	127.1 (2)	N2B—C14B—C15B	126.2 (2)
N2A—C14A—H14A	116.5	N2B—C14B—H14B	116.9
C15A—C14A—H14A	116.5	C15B—C14B—H14B	116.9
C20A—C15A—C16A	120.3 (2)	C20B—C15B—C16B	120.4 (2)
C20A—C15A—C14A	121.7 (2)	C20B—C15B—C14B	123.2 (2)
C16A—C15A—C14A	117.8 (2)	C16B—C15B—C14B	116.4 (2)
C17A—C16A—C15A	121.9 (2)	C17B—C16B—C15B	121.3 (2)
C17A—C16A—H16A	119.1	C17B—C16B—H16B	119.4
C15A—C16A—H16A	119.1	C15B—C16B—H16B	119.4
C16A—C17A—C18A	116.2 (2)	C16B—C17B—C18B	117.1 (2)
C16A—C17A—C33A	124.4 (2)	C16B—C17B—C33B	122.9 (2)
C18A—C17A—C33A	119.4 (2)	C18B—C17B—C33B	120.0 (2)
C19A—C18A—C17A	125.4 (2)	C19B—C18B—C17B	124.7 (2)
C19A—C18A—H18A	117.3	C19B—C18B—H18B	117.7
C17A—C18A—H18A	117.3	C17B—C18B—H18B	117.7
C18A—C19A—C20A	117.6 (2)	C18B—C19B—C20B	117.2 (2)
C18A—C19A—C29A	121.7 (2)	C18B—C19B—C29B	121.6 (2)
C20A—C19A—C29A	120.6 (2)	C20B—C19B—C29B	121.3 (2)
O2A—C20A—C15A	121.5 (2)	O2B—C20B—C15B	121.5 (2)
O2A—C20A—C19A	119.8 (2)	O2B—C20B—C19B	119.3 (2)
C15A—C20A—C19A	118.6 (2)	C15B—C20B—C19B	119.2 (2)
C22A—C21A—C23A	107.9 (2)	C22B—C21B—C24B	106.5 (2)
C22A—C21A—C24A	107.2 (2)	C22B—C21B—C2B	112.2 (2)
C23A—C21A—C24A	110.0 (3)	C24B—C21B—C2B	109.8 (2)
C22A—C21A—C2A	111.3 (2)	C22B—C21B—C23B	108.0 (2)
C23A—C21A—C2A	110.0 (2)	C24B—C21B—C23B	110.2 (2)
C24A—C21A—C2A	110.5 (2)	C2B—C21B—C23B	110.0 (2)
C21A—C22A—H22A	109.5	C21B—C22B—H22D	109.5
C21A—C22A—H22B	109.5	C21B—C22B—H22E	109.5
H22A—C22A—H22B	109.5	H22D—C22B—H22E	109.5
C21A—C22A—H22C	109.5	C21B—C22B—H22F	109.5
H22A—C22A—H22C	109.5	H22D—C22B—H22F	109.5
H22B—C22A—H22C	109.5	H22E—C22B—H22F	109.5
C21A—C23A—H23A	109.5	C21B—C23B—H23D	109.5
C21A—C23A—H23B	109.5	C21B—C23B—H23E	109.5
H23A—C23A—H23B	109.5	H23D—C23B—H23E	109.5
C21A—C23A—H23C	109.5	C21B—C23B—H23F	109.5
H23A—C23A—H23C	109.5	H23D—C23B—H23F	109.5
H23B—C23A—H23C	109.5	H23E—C23B—H23F	109.5
C21A—C24A—H24A	109.5	C21B—C24B—H24D	109.5
C21A—C24A—H24B	109.5	C21B—C24B—H24E	109.5
H24A—C24A—H24B	109.5	H24D—C24B—H24E	109.5
C21A—C24A—H24C	109.5	C21B—C24B—H24F	109.5
H24A—C24A—H24C	109.5	H24D—C24B—H24F	109.5
H24B—C24A—H24C	109.5	H24E—C24B—H24F	109.5
C26A—C25A—C27A	108.5 (3)	C26B—C25B—C4B	109.3 (2)
C26A—C25A—C28A	108.9 (3)	C26B—C25B—C28B	108.2 (2)
C27A—C25A—C28A	107.5 (2)	C4B—C25B—C28B	112.1 (2)
C26A—C25A—C4A	109.4 (2)	C26B—C25B—C27B	109.1 (2)
C27A—C25A—C4A	111.6 (2)	C4B—C25B—C27B	109.4 (2)
C28A—C25A—C4A	110.9 (2)	C28B—C25B—C27B	108.6 (2)
C25A—C26A—H26A	109.5	C25B—C26B—H26D	109.5
C25A—C26A—H26B	109.5	C25B—C26B—H26E	109.5
H26A—C26A—H26B	109.5	H26D—C26B—H26E	109.5
C25A—C26A—H26C	109.5	C25B—C26B—H26F	109.5
H26A—C26A—H26C	109.5	H26D—C26B—H26F	109.5
H26B—C26A—H26C	109.5	H26E—C26B—H26F	109.5
C25A—C27A—H27A	109.5	C25B—C27B—H27D	109.5
C25A—C27A—H27B	109.5	C25B—C27B—H27E	109.5
H27A—C27A—H27B	109.5	H27D—C27B—H27E	109.5
C25A—C27A—H27C	109.5	C25B—C27B—H27F	109.5
H27A—C27A—H27C	109.5	H27D—C27B—H27F	109.5
H27B—C27A—H27C	109.5	H27E—C27B—H27F	109.5
C25A—C28A—H28A	109.5	C25B—C28B—H28D	109.5
C25A—C28A—H28B	109.5	C25B—C28B—H28E	109.5
H28A—C28A—H28B	109.5	H28D—C28B—H28E	109.5
C25A—C28A—H28C	109.5	C25B—C28B—H28F	109.5
H28A—C28A—H28C	109.5	H28D—C28B—H28F	109.5
H28B—C28A—H28C	109.5	H28E—C28B—H28F	109.5
C32A—C29A—C30A	108.2 (2)	C19B—C29B—C32B	110.3 (2)
C32A—C29A—C19A	109.4 (2)	C19B—C29B—C30B	111.8 (2)
C30A—C29A—C19A	111.4 (2)	C32B—C29B—C30B	107.0 (2)
C32A—C29A—C31A	109.1 (2)	C19B—C29B—C31B	109.2 (2)
C30A—C29A—C31A	107.3 (2)	C32B—C29B—C31B	110.3 (2)
C19A—C29A—C31A	111.4 (2)	C30B—C29B—C31B	108.1 (2)
C29A—C30A—H30A	109.5	C29B—C30B—H30D	109.5
C29A—C30A—H30B	109.5	C29B—C30B—H30E	109.5
H30A—C30A—H30B	109.5	H30D—C30B—H30E	109.5
C29A—C30A—H30C	109.5	C29B—C30B—H30F	109.5
H30A—C30A—H30C	109.5	H30D—C30B—H30F	109.5
H30B—C30A—H30C	109.5	H30E—C30B—H30F	109.5
C29A—C31A—H31A	109.5	C29B—C31B—H31D	109.5
C29A—C31A—H31B	109.5	C29B—C31B—H31E	109.5
H31A—C31A—H31B	109.5	H31D—C31B—H31E	109.5
C29A—C31A—H31C	109.5	C29B—C31B—H31F	109.5
H31A—C31A—H31C	109.5	H31D—C31B—H31F	109.5
H31B—C31A—H31C	109.5	H31E—C31B—H31F	109.5
C29A—C32A—H32A	109.5	C29B—C32B—H32D	109.5
C29A—C32A—H32B	109.5	C29B—C32B—H32E	109.5
H32A—C32A—H32B	109.5	H32D—C32B—H32E	109.5
C29A—C32A—H32C	109.5	C29B—C32B—H32F	109.5
H32A—C32A—H32C	109.5	H32D—C32B—H32F	109.5
H32B—C32A—H32C	109.5	H32E—C32B—H32F	109.5
C35A—C33A—C36A	108.3 (2)	C34B—C33B—C35B	110.1 (3)
C35A—C33A—C17A	112.1 (2)	C34B—C33B—C17B	109.1 (2)
C36A—C33A—C17A	108.9 (2)	C35B—C33B—C17B	110.9 (2)
C35A—C33A—C34A	108.5 (3)	C34B—C33B—C36B	107.8 (3)
C36A—C33A—C34A	109.0 (2)	C35B—C33B—C36B	107.4 (3)
C17A—C33A—C34A	110.0 (2)	C17B—C33B—C36B	111.4 (2)
C33A—C34A—H34A	109.5	C33B—C34B—H34D	109.5
C33A—C34A—H34B	109.5	C33B—C34B—H34E	109.5
H34A—C34A—H34B	109.5	H34D—C34B—H34E	109.5
C33A—C34A—H34C	109.5	C33B—C34B—H34F	109.5
H34A—C34A—H34C	109.5	H34D—C34B—H34F	109.5
H34B—C34A—H34C	109.5	H34E—C34B—H34F	109.5
C33A—C35A—H35A	109.5	C33B—C35B—H35D	109.5
C33A—C35A—H35B	109.5	C33B—C35B—H35E	109.5
H35A—C35A—H35B	109.5	H35D—C35B—H35E	109.5
C33A—C35A—H35C	109.5	C33B—C35B—H35F	109.5
H35A—C35A—H35C	109.5	H35D—C35B—H35F	109.5
H35B—C35A—H35C	109.5	H35E—C35B—H35F	109.5
C33A—C36A—H36A	109.5	C33B—C36B—H36D	109.5
C33A—C36A—H36B	109.5	C33B—C36B—H36E	109.5
H36A—C36A—H36B	109.5	H36D—C36B—H36E	109.5
C33A—C36A—H36C	109.5	C33B—C36B—H36F	109.5
H36A—C36A—H36C	109.5	H36D—C36B—H36F	109.5
H36B—C36A—H36C	109.5	H36E—C36B—H36F	109.5
			
O2A—Mn1A—O1A—C1A	−165.9 (2)	O2B—Mn1B—O1B—C1B	−179.6 (2)
N2A—Mn1A—O1A—C1A	−75.6 (4)	N2B—Mn1B—O1B—C1B	−87.4 (3)
N1A—Mn1A—O1A—C1A	−10.0 (2)	N1B—Mn1B—O1B—C1B	−17.7 (2)
Cl1A—Mn1A—O1A—C1A	88.9 (2)	Cl1B—Mn1B—O1B—C1B	78.5 (2)
O1A—Mn1A—O2A—C20A	−168.5 (2)	O1B—Mn1B—O2B—C20B	171.5 (2)
N2A—Mn1A—O2A—C20A	28.8 (2)	N2B—Mn1B—O2B—C20B	14.3 (2)
N1A—Mn1A—O2A—C20A	98.7 (3)	N1B—Mn1B—O2B—C20B	81.2 (4)
Cl1A—Mn1A—O2A—C20A	−66.5 (2)	Cl1B—Mn1B—O2B—C20B	−81.8 (2)
O1A—Mn1A—N1A—C7A	6.3 (2)	O1B—Mn1B—N1B—C7B	18.8 (2)
O2A—Mn1A—N1A—C7A	99.0 (3)	O2B—Mn1B—N1B—C7B	109.0 (3)
N2A—Mn1A—N1A—C7A	170.4 (2)	N2B—Mn1B—N1B—C7B	177.2 (2)
Cl1A—Mn1A—N1A—C7A	−95.5 (2)	Cl1B—Mn1B—N1B—C7B	−87.7 (2)
O1A—Mn1A—N1A—C8A	−174.62 (17)	O1B—Mn1B—N1B—C8B	−168.09 (19)
O2A—Mn1A—N1A—C8A	−81.9 (3)	O2B—Mn1B—N1B—C8B	−77.9 (3)
N2A—Mn1A—N1A—C8A	−10.50 (17)	N2B—Mn1B—N1B—C8B	−9.67 (18)
Cl1A—Mn1A—N1A—C8A	83.59 (16)	Cl1B—Mn1B—N1B—C8B	85.42 (18)
O1A—Mn1A—N2A—C14A	−110.8 (3)	O1B—Mn1B—N2B—C14B	−99.4 (3)
O2A—Mn1A—N2A—C14A	−20.4 (2)	O2B—Mn1B—N2B—C14B	−7.5 (2)
N1A—Mn1A—N2A—C14A	−177.6 (2)	N1B—Mn1B—N2B—C14B	−170.7 (2)
Cl1A—Mn1A—N2A—C14A	84.4 (2)	Cl1B—Mn1B—N2B—C14B	94.2 (2)
O1A—Mn1A—N2A—C13A	75.9 (3)	O1B—Mn1B—N2B—C13B	82.0 (3)
O2A—Mn1A—N2A—C13A	166.31 (17)	O2B—Mn1B—N2B—C13B	173.94 (18)
N1A—Mn1A—N2A—C13A	9.11 (17)	N1B—Mn1B—N2B—C13B	10.74 (18)
Cl1A—Mn1A—N2A—C13A	−88.84 (16)	Cl1B—Mn1B—N2B—C13B	−84.38 (17)
Mn1A—O1A—C1A—C2A	−172.34 (18)	Mn1B—O1B—C1B—C6B	8.4 (4)
Mn1A—O1A—C1A—C6A	8.5 (4)	Mn1B—O1B—C1B—C2B	−174.36 (19)
O1A—C1A—C2A—C3A	−177.7 (2)	O1B—C1B—C2B—C3B	179.0 (2)
C6A—C1A—C2A—C3A	1.6 (4)	C6B—C1B—C2B—C3B	−3.7 (4)
O1A—C1A—C2A—C21A	2.2 (4)	O1B—C1B—C2B—C21B	−2.4 (4)
C6A—C1A—C2A—C21A	−178.5 (2)	C6B—C1B—C2B—C21B	174.9 (2)
C1A—C2A—C3A—C4A	−0.1 (4)	C1B—C2B—C3B—C4B	2.4 (4)
C21A—C2A—C3A—C4A	−180.0 (3)	C21B—C2B—C3B—C4B	−176.2 (3)
C2A—C3A—C4A—C5A	−1.1 (4)	C2B—C3B—C4B—C5B	−0.4 (4)
C2A—C3A—C4A—C25A	175.0 (3)	C2B—C3B—C4B—C25B	177.9 (3)
C3A—C4A—C5A—C6A	0.8 (4)	C3B—C4B—C5B—C6B	−0.3 (4)
C25A—C4A—C5A—C6A	−175.3 (2)	C25B—C4B—C5B—C6B	−178.5 (3)
C4A—C5A—C6A—C7A	178.2 (2)	O1B—C1B—C6B—C7B	6.5 (4)
C4A—C5A—C6A—C1A	0.7 (4)	C2B—C1B—C6B—C7B	−170.7 (3)
O1A—C1A—C6A—C5A	177.3 (2)	O1B—C1B—C6B—C5B	−179.6 (2)
C2A—C1A—C6A—C5A	−1.9 (4)	C2B—C1B—C6B—C5B	3.2 (4)
O1A—C1A—C6A—C7A	−0.1 (4)	C4B—C5B—C6B—C7B	173.0 (3)
C2A—C1A—C6A—C7A	−179.3 (2)	C4B—C5B—C6B—C1B	−1.2 (4)
C8A—N1A—C7A—C6A	179.3 (2)	C8B—N1B—C7B—C6B	175.1 (3)
Mn1A—N1A—C7A—C6A	−1.6 (4)	Mn1B—N1B—C7B—C6B	−12.3 (4)
C5A—C6A—C7A—N1A	179.5 (2)	C1B—C6B—C7B—N1B	−3.6 (5)
C1A—C6A—C7A—N1A	−3.0 (4)	C5B—C6B—C7B—N1B	−177.8 (3)
C7A—N1A—C8A—C13A	−170.7 (2)	C7B—N1B—C8B—C9B	−0.3 (4)
Mn1A—N1A—C8A—C13A	10.1 (3)	Mn1B—N1B—C8B—C9B	−173.7 (2)
C7A—N1A—C8A—C9A	9.8 (4)	C7B—N1B—C8B—C13B	−179.9 (3)
Mn1A—N1A—C8A—C9A	−169.4 (2)	Mn1B—N1B—C8B—C13B	6.8 (3)
C13A—C8A—C9A—C10A	2.2 (4)	C13B—C8B—C9B—C10B	−2.0 (4)
N1A—C8A—C9A—C10A	−178.3 (2)	N1B—C8B—C9B—C10B	178.5 (3)
C8A—C9A—C10A—C11A	−0.6 (4)	C8B—C9B—C10B—C11B	−0.8 (4)
C9A—C10A—C11A—C12A	−0.1 (4)	C9B—C10B—C11B—C12B	2.4 (4)
C10A—C11A—C12A—C13A	−0.7 (4)	C10B—C11B—C12B—C13B	−1.0 (4)
C9A—C8A—C13A—C12A	−3.0 (4)	C11B—C12B—C13B—C8B	−1.8 (4)
N1A—C8A—C13A—C12A	177.5 (2)	C11B—C12B—C13B—N2B	179.0 (3)
C9A—C8A—C13A—N2A	176.9 (2)	C9B—C8B—C13B—C12B	3.3 (4)
N1A—C8A—C13A—N2A	−2.6 (3)	N1B—C8B—C13B—C12B	−177.1 (2)
C11A—C12A—C13A—C8A	2.2 (4)	C9B—C8B—C13B—N2B	−177.4 (2)
C11A—C12A—C13A—N2A	−177.7 (2)	N1B—C8B—C13B—N2B	2.2 (4)
C14A—N2A—C13A—C8A	−179.5 (2)	C14B—N2B—C13B—C12B	−9.5 (4)
Mn1A—N2A—C13A—C8A	−6.2 (3)	Mn1B—N2B—C13B—C12B	169.1 (2)
C14A—N2A—C13A—C12A	0.4 (4)	C14B—N2B—C13B—C8B	171.2 (2)
Mn1A—N2A—C13A—C12A	173.7 (2)	Mn1B—N2B—C13B—C8B	−10.1 (3)
C13A—N2A—C14A—C15A	−179.0 (2)	C13B—N2B—C14B—C15B	−179.8 (3)
Mn1A—N2A—C14A—C15A	8.4 (4)	Mn1B—N2B—C14B—C15B	1.7 (4)
N2A—C14A—C15A—C20A	6.4 (4)	N2B—C14B—C15B—C20B	2.1 (4)
N2A—C14A—C15A—C16A	−178.7 (3)	N2B—C14B—C15B—C16B	−179.5 (3)
C20A—C15A—C16A—C17A	−0.9 (4)	C20B—C15B—C16B—C17B	−0.1 (4)
C14A—C15A—C16A—C17A	−175.9 (2)	C14B—C15B—C16B—C17B	−178.6 (3)
C15A—C16A—C17A—C18A	−1.4 (4)	C15B—C16B—C17B—C18B	−3.0 (4)
C15A—C16A—C17A—C33A	175.9 (2)	C15B—C16B—C17B—C33B	174.1 (3)
C16A—C17A—C18A—C19A	2.8 (4)	C16B—C17B—C18B—C19B	2.0 (4)
C33A—C17A—C18A—C19A	−174.7 (3)	C33B—C17B—C18B—C19B	−175.3 (3)
C17A—C18A—C19A—C20A	−1.6 (4)	C17B—C18B—C19B—C20B	2.2 (4)
C17A—C18A—C19A—C29A	176.1 (3)	C17B—C18B—C19B—C29B	−179.0 (3)
Mn1A—O2A—C20A—C15A	−23.0 (4)	Mn1B—O2B—C20B—C15B	−14.5 (4)
Mn1A—O2A—C20A—C19A	160.04 (18)	Mn1B—O2B—C20B—C19B	166.7 (2)
C16A—C15A—C20A—O2A	−174.8 (2)	C16B—C15B—C20B—O2B	−174.5 (2)
C14A—C15A—C20A—O2A	−0.1 (4)	C14B—C15B—C20B—O2B	3.9 (4)
C16A—C15A—C20A—C19A	2.2 (4)	C16B—C15B—C20B—C19B	4.3 (4)
C14A—C15A—C20A—C19A	176.9 (2)	C14B—C15B—C20B—C19B	−177.3 (3)
C18A—C19A—C20A—O2A	176.1 (2)	C18B—C19B—C20B—O2B	173.6 (2)
C29A—C19A—C20A—O2A	−1.6 (4)	C29B—C19B—C20B—O2B	−5.2 (4)
C18A—C19A—C20A—C15A	−0.9 (4)	C18B—C19B—C20B—C15B	−5.2 (4)
C29A—C19A—C20A—C15A	−178.7 (2)	C29B—C19B—C20B—C15B	175.9 (3)
C3A—C2A—C21A—C22A	−0.1 (4)	C3B—C2B—C21B—C22B	0.8 (4)
C1A—C2A—C21A—C22A	−179.9 (3)	C1B—C2B—C21B—C22B	−177.7 (3)
C3A—C2A—C21A—C23A	119.4 (3)	C3B—C2B—C21B—C24B	119.0 (3)
C1A—C2A—C21A—C23A	−60.5 (3)	C1B—C2B—C21B—C24B	−59.5 (3)
C3A—C2A—C21A—C24A	−119.0 (3)	C3B—C2B—C21B—C23B	−119.5 (3)
C1A—C2A—C21A—C24A	61.1 (3)	C1B—C2B—C21B—C23B	62.0 (3)
C5A—C4A—C25A—C26A	92.4 (3)	C5B—C4B—C25B—C26B	−117.2 (3)
C3A—C4A—C25A—C26A	−83.5 (3)	C3B—C4B—C25B—C26B	64.6 (3)
C5A—C4A—C25A—C27A	−27.6 (4)	C5B—C4B—C25B—C28B	2.7 (4)
C3A—C4A—C25A—C27A	156.5 (3)	C3B—C4B—C25B—C28B	−175.4 (2)
C5A—C4A—C25A—C28A	−147.4 (3)	C5B—C4B—C25B—C27B	123.3 (3)
C3A—C4A—C25A—C28A	36.6 (3)	C3B—C4B—C25B—C27B	−54.8 (3)
C18A—C19A—C29A—C32A	−110.7 (3)	C18B—C19B—C29B—C32B	125.7 (3)
C20A—C19A—C29A—C32A	67.0 (3)	C20B—C19B—C29B—C32B	−55.5 (3)
C18A—C19A—C29A—C30A	8.9 (4)	C18B—C19B—C29B—C30B	6.8 (4)
C20A—C19A—C29A—C30A	−173.4 (2)	C20B—C19B—C29B—C30B	−174.4 (3)
C18A—C19A—C29A—C31A	128.7 (3)	C18B—C19B—C29B—C31B	−112.9 (3)
C20A—C19A—C29A—C31A	−53.7 (3)	C20B—C19B—C29B—C31B	65.9 (3)
C16A—C17A—C33A—C35A	0.4 (4)	C16B—C17B—C33B—C34B	−97.1 (3)
C18A—C17A—C33A—C35A	177.6 (2)	C18B—C17B—C33B—C34B	79.9 (3)
C16A—C17A—C33A—C36A	−119.5 (3)	C16B—C17B—C33B—C35B	24.3 (4)
C18A—C17A—C33A—C36A	57.8 (3)	C18B—C17B—C33B—C35B	−158.6 (3)
C16A—C17A—C33A—C34A	121.2 (3)	C16B—C17B—C33B—C36B	143.9 (3)
C18A—C17A—C33A—C34A	−61.6 (3)	C18B—C17B—C33B—C36B	−39.0 (4)

### Hydrogen-bond geometry (Å, °)

**Table:**

*D*—H···*A*	*D*—H	H···*A*	*D*···*A*	*D*—H···*A*
C7A—H7A···Cl1A^i^	0.93	2.81	3.648 (3)	151
C23A—H23A···O1A	0.96	2.32	2.963 (4)	124
C24A—H24A···O1A	0.96	2.38	3.021 (4)	124
C31A—H31B···O2A	0.96	2.25	2.914 (3)	125
C32A—H32C···O2A	0.96	2.46	3.079 (4)	122
C14B—H14B···Cl1B^ii^	0.93	2.79	3.624 (3)	150
C23B—H23E···O1B	0.96	2.35	2.994 (4)	124
C24B—H24F···O1B	0.96	2.33	2.982 (4)	125
C31B—H31D···O2B	0.96	2.40	3.035 (4)	124
C32B—H32F···O2B	0.96	2.29	2.957 (4)	126
C28A—H28B···Cg1^iii^	0.96	2.70	3.658 (4)	174
C36B—H36D···Cg2^iv^	0.96	2.64	3.597 (4)	174

Symmetry codes: (i) −*x*, −*y*, −*z*+1; (ii) −*x*+1, −*y*, −*z*; (iii) *x*−1, *y*, *z*+1; (iv) *x*, *y*, *z*−1.
